# Supplementary material for: Complexome profiling of the Chlamydomonas psb28 mutant reveals TEF5 as an early PSII assembly factor
Source: Plant Cell. 2025 Mar 17;37(6):koaf055. doi: 10.1093/plcell/koaf055 (PMC12164590; doi:10.1093/plcell/koaf055)
Supplement: koaf055_Supplementary_Data [file koaf055_supplementary_data.zip › psb28_interactive.zip!/plots/intensity/qualityPlots/peptides/PRPS15_Cre06.g264300.t1.2.html]

### Cre06.g264300.t1.2

Trivial name: PRPS15  
SeqCoverage [median]: 18.40  
Mass: 15.79 kDa  
  
 

| Sequence | MissedCleavage | Mass [Da] | wtIntensity | muIntensity | Score |
| --- | --- | --- | --- | --- | --- |
| GLEAILSQR | 0 | 985.56 | 703.06 | 2272.71 | 63.48 |
| NQENINLEK | 0 | 1100.55 | 0.00 | 409.48 | 87.81 |
| SVIVGDTR | 0 | 845.46 | 0.00 | 988.34 | 71.50 |
|  |  |  | 703.1 | 3670.5 |  |
